# Preductal Hemodynamic Redistribution in Preterm Infants with Patent Ductus Arteriosus

**DOI:** 10.1155/2024/7239698

**Published:** 2024-07-24

**Authors:** Aimann Surak

**Affiliations:** Department of Pediatrics University of Alberta, Edmonton, Canada

## Abstract

A patent ductus arteriosus is a common entity in preterm infants. Literature is lacking regarding physiological effects on preductal circulation. This report describes 3 patients with abnormal flow Doppler pattern in brachiocephalic artery. Further research is warranted to better understand the impact of PDA on preductal circulation.

## 1. Introduction

A patent ductus arteriosus (PDA) is a common entity in preterm infants. Unlike in the term population, 57–70% of preterm infants weighing less than 1000 grams will still have an open PDA at 7–10 days of age [1]. Numerous studies described the physiological effects of the PDA, particularly a hemodynamically significant one. When hemodynamically significant, the immature pulmonary vascular bed in this population is exposed to variable degrees of volume overload. This contributes to neonatal morbidities including bronchopulmonary dysplasia, pulmonary haemorrhage, necrotizing enterocolitis, and life-threatening complications [2–5].

PDA is also associated with extra-pulmonary morbidities [5–7]. Historically, it was thought that preductal vessels were protected from the effects of left-to-right ductal shunting. This theory, however, is being re-examined. Lemmers et al. suggest that a persistent PDA may influence brain growth because of insufficient cerebral oxygenation thus affecting neurodevelopmental outcomes [8]. Breatnach et al. found that the brachiocephalic artery tended to have reversal of flow in infants with a PDA at 5 to 7 days of age, whereas those without a PDA had forward flow in this artery postulating that reversal of flow may be an early indicator of impaired cerebral circulation [9]. Sellmer et al. showed that a large PDA early on is associated with a 6-fold increase in the development of an intraventricular haemorrhage (IVH) [10].

The definition of hemodynamically significant PDA has not been standardized [11, 12]. However, it appears that detailed echocardiographic assessment looking at multiple indices is the key to assessing the hemodynamic significance using TnECHO.

## 2. Case Report

**Figure 1:**
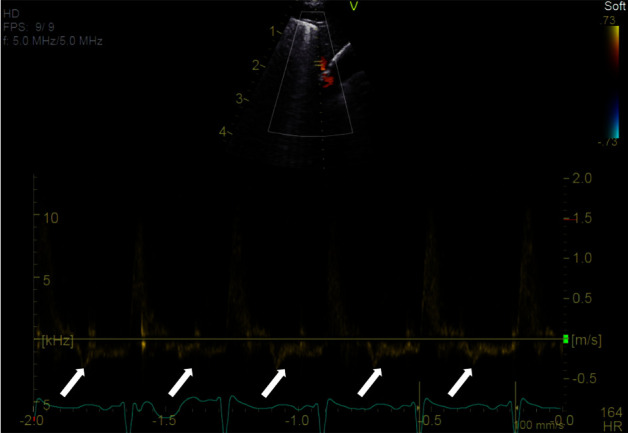
Reversal diastolic flow in brachiocephalic artery (white arrows indicate the reversal of diastolic flow).

We are reporting 3 cases of preterm infants with hemodynamically significant PDAs confirmed by targeted neonatal echocardiogram (TnECHO), presenting with abnormal brachiocephalic artery flow Doppler pattern.  Table 1 summarizes the patients' clinical characteristics, and Table 2 summarizes their PDA characteristics. From the arch view, the brachiocephalic artery was visualized and Dopplered during the echocardiogram. Each of these patients had abnormal Doppler flow pattern in the brachiocephalic artery shown as reversal of diastolic flow (Figure 1).

## 3. Discussion

In this clinical case, we report on three preterm infants with hemodynamically significant PDAs beyond the first week of life. All three infants were noted to have a reversal of diastolic blood flow in the brachiocephalic artery upon assessment by TnECHO, and all were found to have varying degrees of intraventricular hemorrhage. Those scans were the first scans for those infants when they were diagnosed with hemodynamically significant PDA with different ages at the diagnosis for the infants. Also, unfortunately, the subsequent follow-up scans did not interrogate the brachiocephalic flow Doppler pattern. Infants developed mild BPD diagnosed at 36 weeks corrected, but all infants came off respiratory support and were discharged home between 38 and 40 weeks corrected gestation. There are no other insignificant morbidities.

Establishing the impact of the PDA and its significance is a comprehensive process combining clinical, radiological, chemical, and echocardiographic assessment. While a causal relationship cannot be established based on this case report, it appears that a hemodynamically significant PDA might be associated with abnormal Doppler flow patterns in the brachiocephalic artery of preterm infants. Whether this is related to flow redistribution or steal phenomenon remains unclear. There is a paucity of literature on this subject, and therefore, further studies investigating the relationship between abnormal brachiocephalic artery Doppler flow, hemodynamically significant PDAs, and the development of IVH are warranted. As the brachiocephalic artery is easily interrogated and easy to Doppler, if abnormal, this should alert clinicians to the possibility of developing an IVH.

## Figures and Tables

**Table 1:** Patients' clinical characteristics.

	Patient #1	Patient #2	Patient #3
Gestation at birth	24 + 2	24 + 2	26 + 3
Birth weight (g)	730	830	790
Age at the time of scan (days)	10 days	8 days	29 days
Hemodynamic status	Stable; wide pulse pressure with no need for inotropic support	Stable; wide pulse pressure with no need for inotropic support	Stable; wide pulse pressure with no need for inotropic support
PDA treatment	Received 2 courses of ibuprofen, subsequently, PDA closed	Received 2 courses of ibuprofen, and 1 course of acetaminophen, subsequently, PDA closed	Received 1 course of ibuprofen, subsequently, PDA closed
Mode of ventilation	Conventional 18/8, FiO2 0.3	HFOV, FiO2 0.26	HFJV, FiO2 0.4
Mean airway pressure	10	8	14
IVH grade	Bilateral grade I diagnosed on DOL # 4 with subsequent hydrocephalus	Bilateral grade III diagnosed on DOL # 3 with subsequent hydrocephalus	Bilateral grade I diagnosed on DOL # 6 with subsequent hydrocephalus

**Table 2:** Echocardiographic assessment of all patients.

	Patient #1	Patient #2	Patient #3
PDA diameter (mm)	2	2.4	2.6
PDA max systolic velocity (cm/sec)	123	95	213
PDA max diastolic velocity (cm/sec)	68	35	107
PDA mean gradient (mmHg)	2.9	1.3	7
LPA end-diastolic velocity (cm/sec)	30	35	57
LPA mean velocity (cm/sec)	60	55	74
PV diastolic velocity (cm/sec)	38	43	75
LA volume index (ml/m2)	141	95	60
LA : AO ratio	2.8	2	2.4
MV E : A ratio	0.88	0.71	0.99
LVED (cm)	1.4	1.5	2.2
LV IVRT (msec)	30	30	40
LV lateral a' TDI (cm/sec)	6.1	5.4	5.5
LVO (ml/kg/min)	260	528	310
SVC flow (ml/kg/min)	70	45	60
LVO: SVC flow	4	11.7	5.1
Abdominal AO Doppler	AEDF	AEDF	AEDF
Celiac artery Doppler	AEDF	AEDF	AEDF
SMA Doppler	AEDF	AEDF	AEDF
MCA Doppler	Forward	Forward	AEDF

## Abbreviations

IVH: Intraventricular haemorrhage
PDA: Patent ductus arteriosus
TnECHO: Targeted neonatal echocardiogram
AEDF: Absent end-diastolic flow.
